# Development and Characterization of Pinecone Syrup-Enriched Turkish Delight: Physicochemical, Antioxidant, Textural, and Sensory Properties

**DOI:** 10.3390/foods15213862

**Published:** 2026-10-28

**Authors:** Nadide Seyhun

**Affiliations:** Department of Food Engineering, Faculty of Engineering and Natural Sciences, Bursa Technical University, Bursa 16310, Türkiye; nadide.seyhun@btu.edu.tr

**Keywords:** black pine, confectionery, lokum, food fortification, herbal syrups, pinecone decoction, pinecone syrup, *Pinus nigra*, plant-derived extracts, Turkish delight

## Abstract

Turkish delight (lokum) is a traditional starch-gel confectionery widely consumed in the Middle East and Mediterranean regions but characterized by limited nutritional value and bioactive content. Pinecone syrup, a traditional herbal syrup used in folk medicine, is recognized for its rich phenolic composition and antioxidant capacity; yet its intense resinous flavor presents an organoleptic barrier to direct consumption. This study evaluated whether the Turkish delight matrix could moderate the sensory impact of sugar-free pinecone syrup, while preserving its texture and increasing its functional potential. Formulations with 0% (control), 50%, and 100% PCS substitution were characterized for physicochemical, colorimetric, textural, antioxidant, and sensory properties. PCS incorporation significantly increased total phenolic content and DPPH radical scavenging activity compared with the control. Color shifted concentration-dependently from yellow to reddish-brown, hardness decreased moderately, and FT-IR suggested alterations in PCS–starch intermolecular interactions. Both PCS formulations received acceptable sensory scores; however, the 50% substitution achieved the highest taste (4.62/5) and overall preference (4.48/5), providing the optimal balance between bioactive enrichment and sensory acceptability. These findings demonstrate the potential of Turkish delight as a confectionery matrix for incorporating pinecone-derived bioactive compounds.

## 1. Introduction

Turkish delight, known as lokum in Turkish, is one of the oldest and most widely recognized confectionery products in the Middle East and Mediterranean regions. With origins dating back several centuries, Turkish delight has maintained a central place in Turkish food culture as well as in Balkan and Middle Eastern countries, and has gained international recognition as a traditional delicacy [1,2]. Turkish delight is a jelly-like confectionery product mainly made of starch and sugar, with other ingredients like citric acid and water [1,3]. It is conventionally produced by gelatinizing starch in a concentrated sugar solution, resulting in a semi-transparent, chewy gel structure with a characteristic soft, elastic and sticky texture [1,2,3,4].

Turkish delight is a versatile confectionery product that can be formulated with a broad range of ingredients such as rose water and petals, several types of nuts, dried fruits, and various natural flavoring agents; although its plain version remains a well-liked and widely consumed product [5,6]. This versatility provides a broad scope for further formulation modifications and the incorporation of novel ingredients to develop innovative Turkish delight products.

Despite their broad appeal and high consumer acceptance driven by attractive sensory qualities, conventional confectionery products—including Turkish delight—are usually nutritionally poor, providing predominantly simple sugars and refined carbohydrates while lacking meaningful concentrations of bioactive constituents with health-promoting potential [7]. The global food industry has increasingly shifted toward the development of functional foods—products that deliver health benefits beyond basic nutrition through the incorporation of probiotics, vitamins, minerals, bioactive compounds, and other naturally occurring substances [8,9]. As health-conscious consumers increasingly seek foods that offer functional benefits alongside sensory satisfaction, the confectionery sector faces increasing pressure to reformulate its products accordingly [10]. In this context, confectionery products have been recognized as a suitable matrix for the delivery of bioactive ingredients, owing to their wide consumption, high consumer acceptance, and capacity to incorporate a diverse range of functional additives without substantially compromising their characteristic sensory properties [7,10]. The incorporation of herbal extracts, plant-derived syrups, and other phytochemical-rich ingredients into confectionery formulations has therefore attracted considerable research interest as a strategy to enhance both the nutritional value and the health-promoting profile of these products [7].

Pinecone syrup (PCS) is a herbal beverage obtained by boiling the cones of trees belonging to the *Pinaceae* (pine family), usually with sugar, until a syrup consistency is achieved. The leaves, bark, cones, and resins of trees belonging to the *Pinaceae* family are believed to have healing effects in folk medicine, and products derived from them are used in the treatment of various ailments, primarily coughs, but also stomach problems, asthma, bronchitis, and other diseases [11,12]. Especially after the COVID-19 pandemic, the demand for pinecone syrup has increased due to its effects on upper respiratory tract illnesses.

The use of PCS for health-related purposes is not unique to Türkiye but is also documented in several European and Asian countries as part of traditional medicinal practices [11,13,14,15]. It has been reported that the PCS obtained from the mountain pine (*Pinus mugo*) in Italy has long been used in folk medicine for sore throat, cough and upper respiratory tract diseases [13,14]. In Latvia, herbal syrups made of pine buds and pinecones are known to be widely used against cough and for their anti-inflammatory and antibacterial effects in traditional medicine [11,15].

Pinecone syrup is recognized for its rich bioactive composition, including phenolic acids and flavonoids with antioxidant properties [11,15,16]. Among herbal syrups, pinecone syrup ranks among the highest in total phenolic content, reaching up to 282 mg GAE/100 g, with correspondingly high radical scavenging activity [11]. However, products made using pinecones are characterized by a specific and intense taste and smell, and even though it is usually prepared with sugar, certain volatile compounds that give a resinous pine aroma are notably intense in PCS [16], presenting a significant organoleptic barrier to direct consumption. Moreover, PCS is predominantly produced by traditional household methods, without standardized protocols or rigorous quality and safety controls. Preparation practices vary considerably between households, regions, and countries with respect to the pine species used, cone maturity, processing conditions, and ingredient ratios, resulting in high batch-to-batch variability in phytochemical composition, safety-relevant parameters, and organoleptic properties [11,12,13,14,15,16]. This variability limits the reproducibility and comparability of findings across studies and underscores the need for systematic scientific investigation before PCS can be considered for broader food applications.

While PCS has traditionally been consumed as a beverage, its potential applications extend well beyond this to include a broader range of food products and confectionery formats including jams, desserts, and sweet matrices. A recent study showed that the incorporation of pinecone-derived ingredients into confectionery products is technically feasible and promising as a strategy to simultaneously satisfy consumer hedonic expectations and meet growing health-oriented demands [16]. Turkish delight, as a dense, sugar-starch matrix with a neutral base flavor and strong capacity to absorb and retain aromatic compounds, offers a promising vehicle for masking the organoleptic intensity of pinecone syrup while delivering its functional benefits in an acceptable format.

Limited information is available on the incorporation of herbal syrups or herbal extracts into Turkish delight and their effects on its properties. Uslu et al. [17] investigated the effects of starch concentration and soapwort extract addition on the physicochemical, textural, and sensory properties of Turkish delight, demonstrating that these formulation variables influenced the final product quality. Duruk [18] reported that the incorporation of herbal teas into Turkish delight formulations modified physicochemical, textural, and sensory properties without compromising overall acceptability, stating the potential of plant-derived ingredients to enhance the functional and sensory profile of Turkish delight. However, to date, no study has investigated the incorporation of pinecone syrup or extract into Turkish delight formulations or characterized the resulting physicochemical, functional, and sensory properties of such a product. This study therefore investigated the effects of replacing water with sugar-free pinecone syrup at two substitution levels, given the growing consumer interest in functional confectionery and the recognized bioactive potential of pinecone-derived ingredients. The aim of this study was to develop Turkish delight formulations incorporating sugar-free pinecone syrup as a functional ingredient and to investigate its effects on the physicochemical, functional, textural, and sensory properties of the resulting products. Turkish delights containing two different levels of pinecone syrup (50% and 100%) were compared with a control formulation to determine the influence of pinecone syrup incorporation on color, total phenolic content, antioxidant activity, texture, and sensory characteristics. In addition, the study aimed to assess the potential of pinecone syrup as a value-added ingredient for the development of a functional Turkish delight product.

## 2. Materials and Methods

### 2.1. Materials

**Figure 1:**
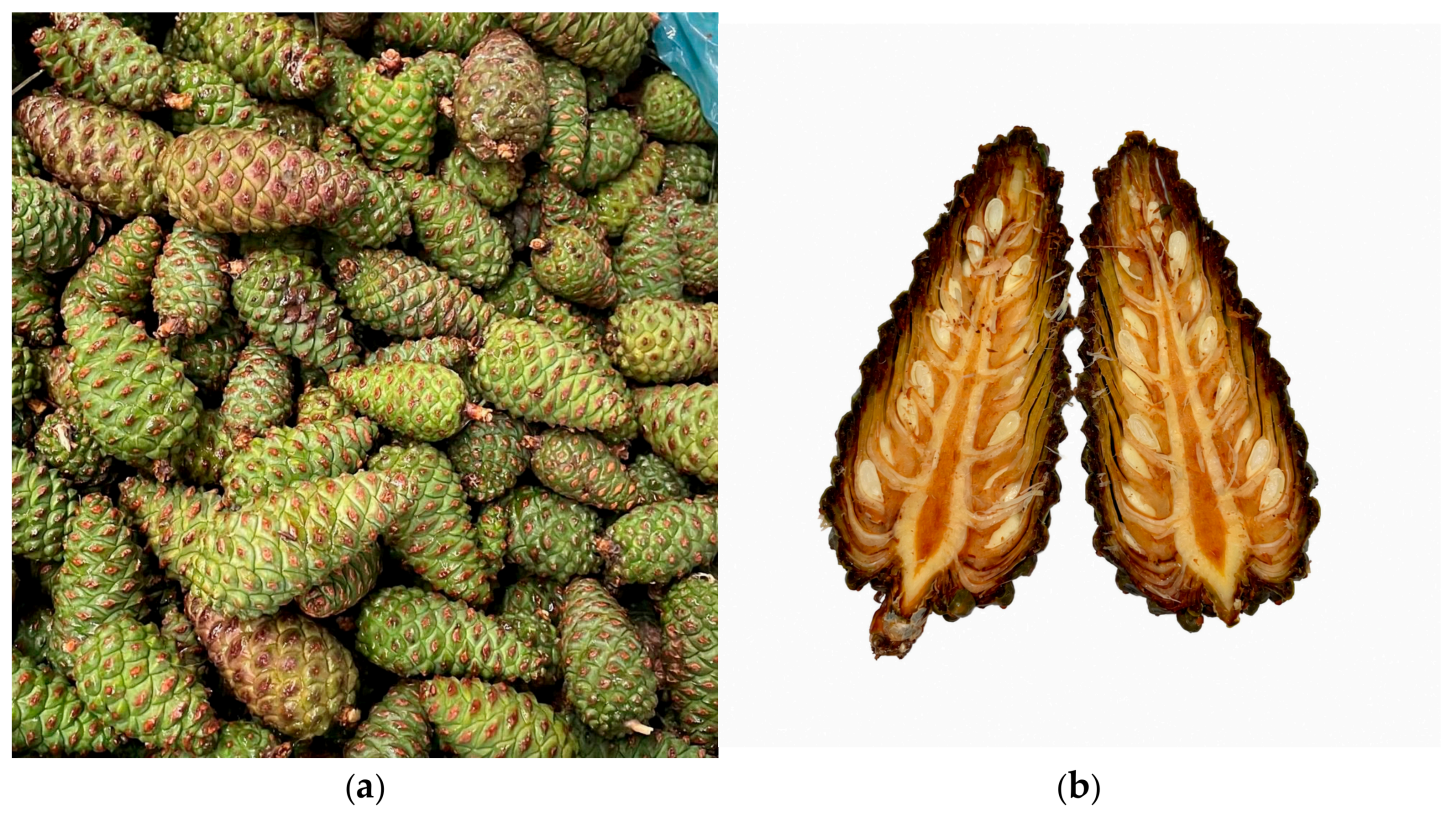
(**a**) Harvested black pine (*Pinus nigra*) cones for the present study. (**b**) The cross-section photo of a black pine (*Pinus nigra*) cone.

Black pine (*Pinus nigra*) cones used for pinecone syrup (PCS) production in this study. The pinecones were harvested when still young from a forest area in the Taurus Mountains near Arslanköy, Mersin, Türkiye (37°01′12″ N 34°17′31″ E) in June 2025, transferred to the laboratory and stored frozen (−20 °C) until further use (Figure 1). The pinecones were collected and identified as *Pinus nigra* by forestry specialists from the Mersin Regional Directorate of Forestry, Türkiye.

Corn starch (Migros, Türkiye), white sugar (Migros, Türkiye) and citric acid (Bağdat, Türkiye) were bought from a local supermarket. All other chemicals and reagents used were of analytical reagent grade.

### 2.2. Methods

#### 2.2.1. Pinecone Syrup Production

The pinecones were thoroughly washed and cut into small pieces. Water was then added at a pinecone-to-water ratio of 1:4 (*w*/*v*), and the mixture was boiled for 4 h. No sugar or other ingredients were added during the production process. The mixture was then filtered through a cheesecloth. The syrup obtained was stored in a refrigerator until use on the same day.

The syrup obtained in this study is indeed an aqueous pinecone extract prepared by decoction rather than a conventional concentrated sugar syrup. The term pinecone syrup (PCS) is retained to reflect commercial usage for pinecone products without added sugar and similar terminology in the literature [19], although its low soluble solids content distinguishes it from conventional concentrated sugar syrups.

#### 2.2.2. Analyses of the Pinecone Syrup

- Physicochemical analyses

The pH values of the samples were measured using a pH meter (Ohaus, ST3100, Nänikon, Switzerland) at room temperature.

The total soluble solids content of the samples was determined using a digital refractometer (KEM, RA-600, Kyoto, Japan). The results were expressed as °Brix.

A moisture analyzer (Ohaus, MB45, Nänikon, Switzerland) was used to determine the moisture content of the samples. The results were listed as percentage moisture content (%).

The water activity (a_w_) of the samples was determined using a water activity meter (Novasina AG, Lachen, Switzerland). The results were expressed as a_w_.

- Color analysis

The color parameters of the samples were determined using a colorimeter (HunterLab, UltraScan VIS, Reston, VA, USA) and expressed in terms of L* (lightness; black (0) to white (100)), a* (red/green axis), b* (yellow/blue axis), C* (chroma; intensity or saturation of the color) and h° (hue angle; the actual color (like red, yellow, green, blue) on a 360-degree wheel) values.

- Total phenolic content and antioxidant activity

The total phenolic content of the samples was determined using the Folin–Ciocalteu method [20]. Briefly, the sample extracts were reacted with Folin–Ciocalteu reagent, followed by the addition of sodium carbonate solution. After being kept in the dark for 2 h, the absorbance was measured with a microplate reader (Epoch Microplate Spectrophotometer, BioTek, Santa Clara, CA, USA) at 760 nm. Gallic acid was used as the calibration standard, and the results were expressed as mg gallic acid equivalents (GAE) per 100 mL of sample.

The antioxidant activity of the samples was evaluated using the 2,2-diphenyl-1-picrylhydrazyl (DPPH) radical scavenging assay [21]. Sample extracts were mixed with DPPH solution and incubated in the dark for 2 h. The absorbance was subsequently measured at 517 nm with a microplate reader (Epoch Microplate Spectrophotometer, BioTek, Santa Clara, CA, USA), and the radical scavenging activity was calculated based on the decrease in absorbance. Trolox was used for the calibration and the results were expressed as μmol TE/100 mL.

The antioxidant activity was also determined using the ABTS (2,2-azino-bis-3-ethylbenzothiazoline-6-sulfonic acid) radical cation scavenging assay [22]. The ABTS radical cation solution was prepared and kept for 16 h at room temperature in the dark. The absorbance was measured at 734 nm with a microplate reader (Epoch Microplate Spectrophotometer, BioTek, Santa Clara, CA, USA), and the antioxidant activity was calculated accordingly. Trolox was used for the calibration curve and the results were given as μmol TE/100 mL.

- HMF analysis

The hydroxymethylfurfural (HMF) content of the samples was determined by high-performance liquid chromatography (HPLC), with modifications to the method described by Tan et al. [23]. Briefly, 5 g of sample was diluted to 50 mL with distilled water, sonicated in an ultrasonic water bath for 1 min, and filtered through a 0.22 µm membrane filter before being transferred into HPLC vials. Chromatographic analysis was performed using an Agilent HPLC system equipped with a diode array detector (DAD) and an Agilent C18 column (5 µm, 4.6 × 250 mm; Agilent, Santa Clara, CA, USA). The column temperature was maintained at 30 °C. Isocratic elution was performed using water and acetonitrile (95:5, *v*/*v*) as the mobile phase at a flow rate of 1.0 mL/min. The injection volume was 20 µL, and HMF was detected at 284 nm. HMF was quantified using a calibration curve covering 0–100 ppm (R^2^ > 0.99), and the results were expressed as mg/kg of sample.

- FT-IR analysis

Fourier Transform Infrared (FT-IR) measurements were performed with an FT-IR spectrometer (Thermo Fisher Scientific Co., Waltham, MA, USA), using wave numbers in the range 400–4000 cm^−1^, with a resolution of 4 cm^−1^.

#### 2.2.3. Turkish Delight Production

**Figure 2:**
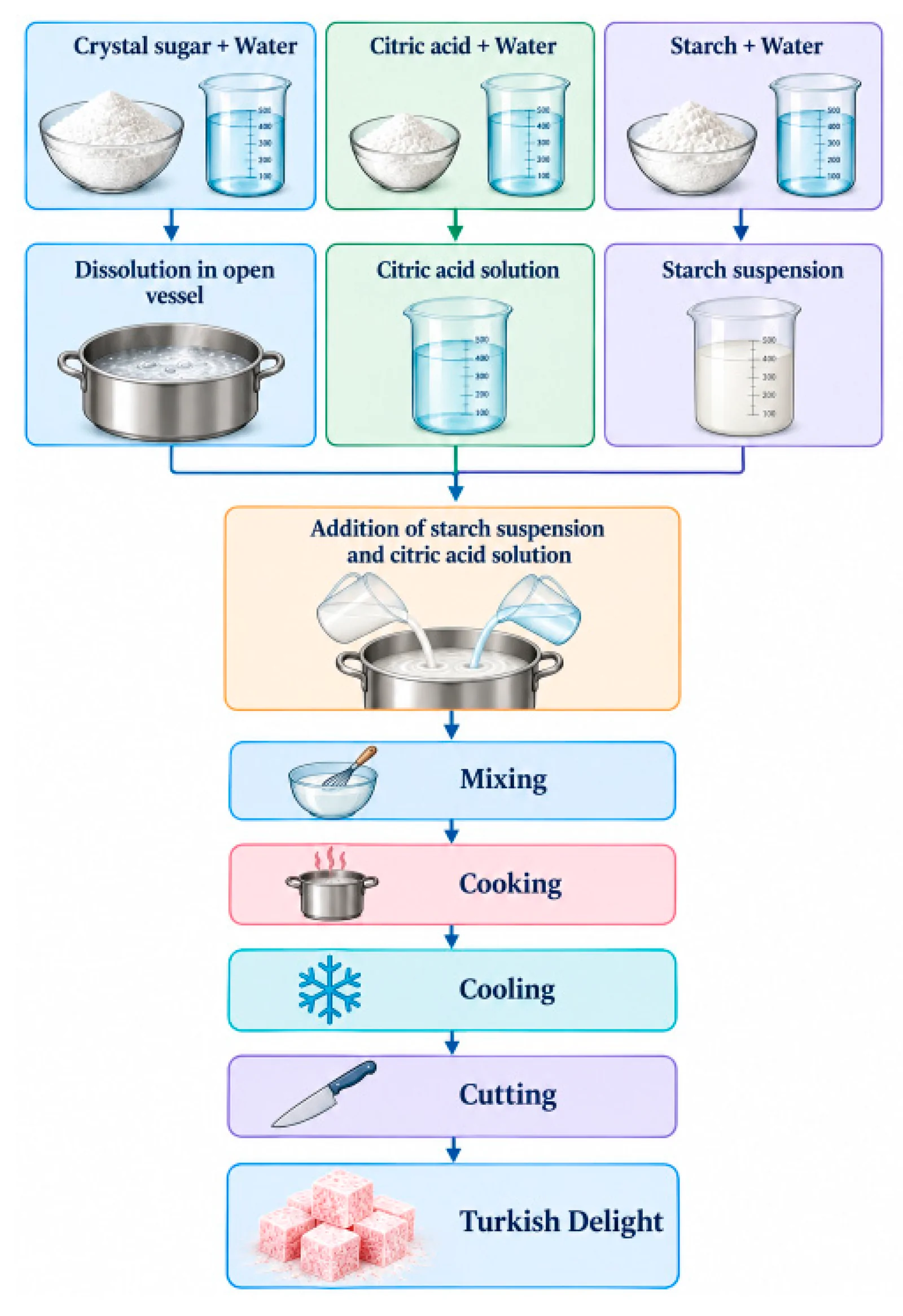
Turkish delight production flowchart.

The Turkish delight formulation and production process were adapted from Duruk [18]. Briefly, white sugar was dissolved in 500 mL of water and transferred to an open vessel. Citric acid was separately dissolved in 100 mL of water, while corn starch was dispersed in 400 mL of water to obtain a homogeneous starch suspension. The starch suspension and citric acid solution were subsequently added to the sugar solution and thoroughly mixed. The mixture was cooked in an open vessel for 100 min under continuous stirring. The cooking time was used as the processing endpoint. Following cooking, the hot mass was transferred into a rectangular tray sprinkled with corn starch and allowed to cool to room temperature, and then cut into 3 × 3 cm pieces. The overall production flowchart is illustrated in Figure 2.

To produce pinecone syrup enriched Turkish delight samples, the water in the formulation was replaced with sugar-free pinecone syrup. The formulations used for the production of the Turkish delights are presented in Table 1.

#### 2.2.4. Analyses of Turkish Delight Samples

- Physicochemical analyses

The pH values of the Turkish delight samples were determined using the method described in Section 2.2.2. Prior to analysis, the samples were homogenized, and the pH measurements were performed at room temperature.

The moisture content, total soluble solids content, and water activity of the Turkish delight samples were determined using the methods described in Section 2.2.2.

The ash content of the samples was determined by the gravimetric incineration method using a muffle furnace (Protherm PLF 110-8, Protherm Furnaces, Ankara, Türkiye). The results were given as percentage ash content (%).

The total sugar content of the samples was determined by the Lane—Eynon titrimetric method. The results were expressed as a percentage (%) of total sugars on a fresh weight basis.

- Color analysis

The color parameters of the samples were determined using the method described in Section 2.2.2. The Turkish delight samples were cut in half with a knife and then the color measurements were performed. The sample that did not contain PCS was used as the reference for total color change (ΔE) calculation. Total color change (ΔE) was calculated as follows:

(1) $$\Delta E=\sqrt{(L_{\mathrm{sample}}^{*}-L_{\mathrm{ref}}^{*}{)}^{2}\;+\;(a_{\mathrm{sample}}^{*}-a_{\mathrm{ref}}^{*}{)}^{2}\;+\;(b_{\mathrm{sample}}^{*}-b_{\mathrm{ref}}^{*}{)}^{2}}$$

- Total phenolic content and antioxidant activity

For the extraction process, 0.5 g of sample was weighed into a Falcon tube, 10 mL of methanol (9:1, methanol–ultra pure water) was added, and the mixture was agitated for 2 min in a mixer (Ultraturrax, IKA, Staufen, Germany), then centrifuged at 3000× *g* for 5 min. The supernatants were transferred into tubes.

The total phenolic content and antioxidant activity of the samples were determined using the methods described in Section 2.2.2. The total phenolic content results were expressed as mg gallic acid equivalents (GAE) per 100 g of sample. The DPPH and ABTS results were expressed as μmol TE/100 g.

- Texture analysis

Texture profile analysis (TPA) of the samples was performed using a texture analyzer (TA-XT Plus, Stable Micro Systems, Godalming, Surrey, UK), equipped with a 35 mm diameter cylindrical probe and a 5 kg load cell, by following the method of Pocan et al. [24] with some modifications. The experiments were performed at room temperature with a compression ratio of 30%.

- HMF analysis

The hydroxymethylfurfural (HMF) content of the samples was determined by high-performance liquid chromatography (HPLC). Briefly, 5 g of sample was weighed and made up to 50 mL with distilled water. The sample was kept in an ultrasonic water bath for 1 min to facilitate extraction. The extract was then filtered through a 0.22 µm membrane filter and transferred into HPLC vials prior to analysis. HMF content of the samples was determined using the method described in Section 2.2.2.

- FT-IR analysis

Fourier Transform Infrared (FT-IR) measurements were performed using the method described in Section 2.2.2.

- Sensory analysis

All sensory evaluation procedures were reviewed and approved by the Science, Engineering and Social Sciences Research Ethics Committee of Bursa Technical University (date of approval: 27 February 2026; meeting no. 2026-4; decision no. 3). Prior to testing, all participants were thoroughly informed regarding the nature of the study, the ingredients used in the formulations, and potential allergen risks. Written informed consent was obtained from each panelist, and participation was entirely voluntary with the right to withdraw at any stage.

Sensory analysis was conducted using a 5-point hedonic scale (1 = dislike extremely, 3 = neither like nor dislike, 5 = like extremely) by an evaluation panel of 25 semi-trained panelists (aged 25–50 years), all of whom were graduate students and academic staff in the food engineering department with basic training in sensory analysis, in line with the assessor categories described in ISO 8586:2023 [25]. Turkish delight samples were brought to ambient temperature and presented in odorless, food-grade containers marked with randomized three-digit codes. Panelists evaluated six primary attributes: color, smell, taste, texture, mouthfeel, and overall preference. To eliminate sensory fatigue and carryover effects, drinking water and unsalted crackers were served between evaluations.

#### 2.2.5. Statistical Analysis

All measurements were performed in triplicate, and the results were expressed as mean ± standard deviation (SD). Statistical analyses were performed using Minitab Statistical Software v.16 (Minitab Inc., State College, PA, USA). The data were subjected to one-way analysis of variance (ANOVA) to determine the significance of differences among the samples. When significant differences were detected, Tukey’s multiple comparison post hoc test was used. Differences were considered statistically significant at *p* < 0.05.

## 3. Results and Discussion

### 3.1. Pinecone Syrup Analyses

The physicochemical, phenolic, antioxidant, HMF, and color characteristics of the pinecone syrup (PCS) are presented in Table 2. The PCS had a moisture content of 97.98 ± 0.19%, a °Brix value of 2.73 ± 0.06, and a water activity of 0.960 ± 0.002. The high moisture content and water activity, together with the low °Brix value, indicate that the PCS used in the present study was highly aqueous. These characteristics can be attributed to the preparation method, in which no sugar was added during syrup production, unlike the formulations reported in other studies [15,16,26], where sugar is commonly used as a main ingredient. Accordingly, the PCS used in this study is better described as a pinecone decoction—an aqueous extract prepared by boiling—rather than a conventional sugar-based syrup.

The PCS exhibited an acidic character with a pH of 3.76 ± 0.01 (Table 2). This result is generally consistent with pH values reported for different pinecone syrups in the literature. For example, the pH of pinecone syrups is reported to range between 3.36–3.96 depending on the preparation method [15], and a recent study comparing different herbal syrups reported a pH of 3.68 ± 0.03 for pinecone syrup [26].

The total phenolic content (TPC) of the PCS was 237.72 ± 15.95 mg GAE/100 mL, while DPPH and ABTS values were 434.78 ± 0.48 μmol TE/100 mL and 157.98 ± 0.19 μmol TE/100 mL, respectively (Table 2). The results showed that PCS exhibited considerable phenolic content and antioxidant activity, meaning that the syrup contained phenolic and other antioxidant-active compounds. Pinecones and cone-derived products have previously been reported as sources of phenolic compounds and antioxidant activity. A recent study reported that the TPC of pinecone syrup made from *Pinus sylvestris* cones was 360.62 ± 8.51 mg GAE/100 g [26], while another study reported that *Pinus sylvestris* cone syrup resulted in TPC values around 250 mg GAE/100 g and DPPH around 125 mg TE/100 g [11]. The antioxidant potential of pinecone-derived syrups may be associated with the extraction of phenolic compounds from the cones during syrup production. However, their antioxidant properties may vary depending on the *Pinus* species used and preparation method. Pinecone syrup is produced by varying methods across different cultures and regions, even households. The species of pine, the maturity and freshness of the cones, the cone-to-water-to-sugar ratio, boiling duration, and filtration conditions all influence the concentration and composition of phenolic compounds extracted into the final syrup [11,15,16,26]. This variability is reflected in the wide range of TPC and antioxidant capacity values reported across studies.

The color parameters were measured as L* = 40.90 ± 0.21, a* = 7.48 ± 0.08, b* = 11.58 ± 0.20, C* = 13.79 ± 0.21, and h° = 57.14 ± 0.20 (Table 2). The color of pinecone syrup exhibited a moderately dark appearance (L* = 40.90), and a hue angle of 57.14 positioned it in the amber-orange region of the CIE color space (45° < h° < 65°). Hue angle (h°) defines the perceived color tone on a circular scale, where values of 0° or 360° correspond to red-purple, 90° to yellow, 180° to green, and 270° to blue. The positive a* and b* values indicate simultaneous red and yellow color contributions, consistent with the amber-orange appearance of PCS.

### 3.2. Turkish Delight Analyses

#### 3.2.1. Physicochemical Analyses

The results of the physicochemical analyses for the Turkish delight samples are given in Table 3.

The pH values of Turkish delight samples decreased significantly with increasing PCS concentration (Table 3). The control sample exhibited the highest pH (4.24 ± 0.13), while the 100% PCS group showed the lowest (3.67 ± 0.02). Tukey’s post hoc test confirmed that the control was significantly different from both PCS groups (*p* < 0.05), whereas the difference between the 50% and 100% PCS groups was not statistically significant. This acidification is consistent with the naturally low pH of PCS itself (pH 3.76, Table 2), which is attributed to the presence of organic acids released during the extraction and boiling process [15].

The pH value of the control sample observed in this study was comparable to those reported for plain Turkish delight in previous studies, which ranged from pH 4.11 [2] and 4.52 [27]. Kavak & Ogun [27] added two different levels (%10 and %15) of quince pulp (pH 3.29) to the Turkish delight formulation to develop a functional confectionery product. The addition of an acidic fruit pulp to Turkish delight resulted in a significant decrease in pH with increasing levels of quince pulp, from 4.52 in the control to 4.07 and 3.93 in the formulations containing 10% and 15% quince pulp, respectively, consistent with the findings of the present study.

There were no statistically significant differences among the three groups for moisture content or water activity, although a declining trend in water activity was noted with increasing PCS concentration. The insignificant moisture differences indicate that PCS substitution did not substantially alter the water-binding capacity of the starch-gel matrix.

Ash content increased significantly with PCS incorporation, with the 100% PCS sample differing significantly from the control sample. The higher ash content of PCS100 indicates a greater proportion of inorganic residue, but does not identify the elements contributing to this increase. İncemehmetoğlu [28] reported ash contents of 0.24–0.96% and considerable variation in mineral composition among traditionally produced pinecone molasses samples. However, differences in formulation and concentration limit direct comparison with the present products. Furthermore, ash content alone cannot establish nutritional benefits or exclude the presence of potentially toxic elements. As elemental composition was not determined in the present study, future research should characterize both PCS and the final confectionery products for nutritionally relevant elements and potentially toxic elements, including lead, cadmium, arsenic, and mercury.

Total sugar content did not differ significantly among the groups, which is consistent with the same amount of sucrose being added to all formulations and the use of PCS prepared without added sugar. The comparable total sugar contents provide useful context for interpreting the sensory results, although sweetness intensity was not evaluated separately.

#### 3.2.2. Color Analysis

**Figure 3:**
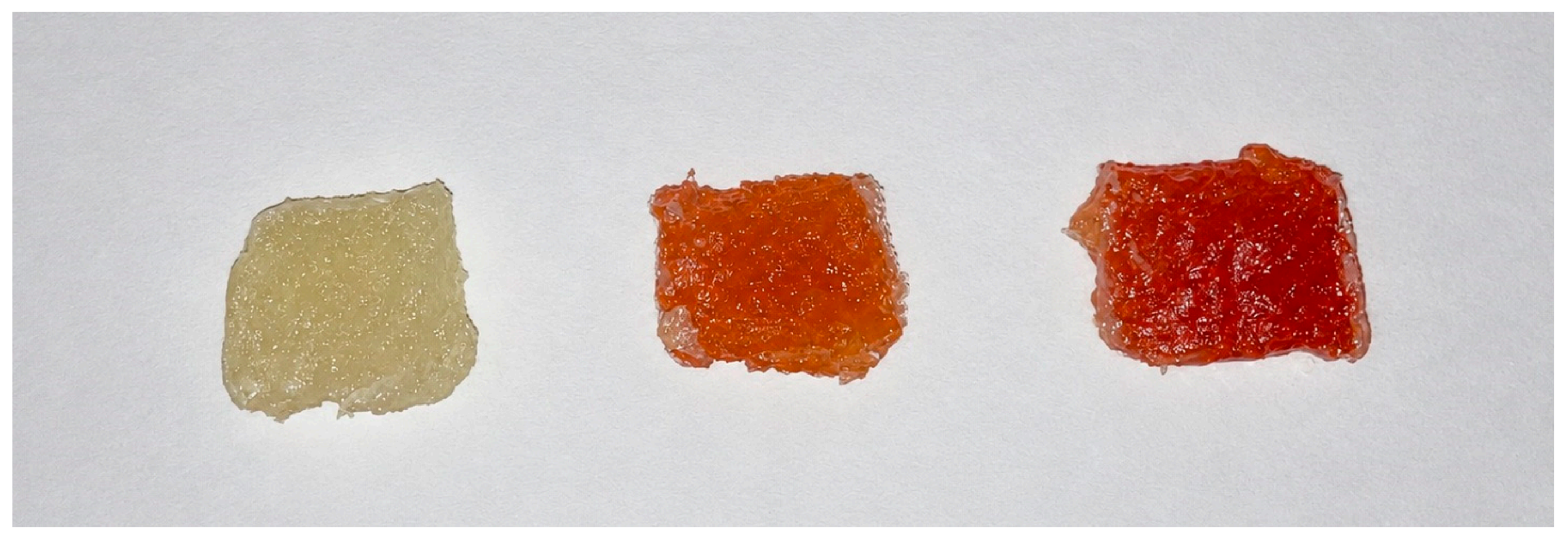
Turkish delight samples (left to right: Control–PCS50–PCS100).

The Turkish delight samples produced for this study are cut in half for color measurements and photographed (Figure 3). Color measurement results are given in Table 4.

Incorporation of PCS into Turkish delight formulation produced significant, concentration-dependent changes in all five CIE color parameters of the final products (Table 4). These visual differences are also clearly evident in Figure 3.

Color is an important quality parameter for Turkish delight, and some confectioners have stated that the typical color of plain Turkish delight should be in the range of white to a golden amber color [29]. A study on developing a functional Turkish delight formulation reported the color values for the plain Turkish delight as L* = 57.32, a* = −1.7, b* = 13.5 [2], which are comparable to those of the control formulation in the present study (L*, a*, b* found as 63.90, −1.78, 12.79, respectively).

Lightness (L*) decreased progressively with increasing PCS concentration, meaning the samples got darker with increasing PCS percentage. This darkening is also reflected in the sample photos (Figure 3). The red–green index (a*) shifted from mildly negative in the control sample (−1.78 ± 0.18), characteristic of plain starch-sugar confectionery, to strongly positive values in both PCS-enriched samples (50% PCS: 12.93 ± 0.31; 100% PCS: 13.97 ± 1.04). The two PCS groups were not significantly different from each other (*p* = 0.191). The 50% PCS sample displayed the highest b* (24.33 ± 1.76), significantly exceeding both the control (12.79 ± 0.90) and the 100% PCS sample (10.44 ± 1.16), which were not significantly different from each other (*p* = 0.155).

Chroma (C*) followed the same pattern, with the 50% PCS formulation yielding the highest color saturation (27.56 ± 1.42), significantly exceeding both the control (12.92 ± 0.88) and the 100% PCS sample (17.44 ± 1.53). Hue angle (h°) decreased across all groups, with all pairwise comparisons highly significant (*p* < 0.001): Control (97.95° ± 1.14°)—50% PCS (61.94° ± 2.23°)—100% PCS (36.71° ± 1.04°). The h° of the PCS (57.14°, Table 2) fell between the two PCS-enriched Turkish delight formulations. The 100% PCS sample (36.71°) exhibited a lower h° than the syrup itself (57.14°), shifting from the amber-orange zone into the reddish-brown zone (h° < 40°).

The total color difference (ΔE*) with respect to the control sample was calculated as 23.11 ± 0.72 and 30.99 ± 0.74 for the 50% and 100% PCS formulations, respectively. The ΔE* value indicates whether the color change is noticeable. If ΔE* > 5, the colors of the samples are considered fundamentally different [30]. The ΔE* values of 50% and 100% PCS formulations confirmed that PCS incorporation produced a readily perceptible and concentration-dependent color change in the final product. These instrumental colorimetric findings were consistent with the sample photographs (Figure 3). Similarly, Batu and Arslan [31] reported that incorporating black grape and sour cherry syrups into Turkish delight decreased L* values, resulting in darker products. This agrees with the reduction in lightness observed following PCS incorporation, although differences in ingredient composition limit direct comparison of the underlying color changes.

#### 3.2.3. Total Phenolic Content and Antioxidant Activity

The total phenolic content and antioxidant capacity results of Turkish delight samples are given in Table 5. PCS incorporation caused a significantly sharp increase in TPC, with all pairwise comparisons highly significant (*p* < 0.001). TPC rose from 7.29 ± 1.07 mg GAE/100 g in the control to 130.06 ± 8.98 mg GAE/100 g in the 50% PCS group and 208.57 ± 5.22 mg GAE/100 g in the 100% PCS group. The PCS itself contained 237.72 ± 15.95 mg GAE/100 mL, while the TPC of the 100% PCS formulation reached 208.57 ± 5.22 mg GAE/100 g, indicating substantial transfer of PCS-derived phenolic constituents into the final product.

The antioxidant activity also increased dramatically with PCS incorporation, as measured by both DPPH and ABTS radical scavenging assays (Table 5). DPPH values rose from 5.06 ± 0.96 μmol TE/100 g in the control to 417.56 ± 9.77 μmol TE/100 g in the 50% PCS group and 435.61 ± 0.87 μmol TE/100 g in the 100% PCS group. Both PCS formulations differed significantly from the control and from each other, indicating a concentration-dependent antioxidant response. For ABTS, both PCS groups (158.52 and 157.66 μmol TE/100 g) were not significantly different from each other, but they both exceeded the control sample (11.31 ± 7.19 μmol TE/100 g).

DPPH activity increased from PCS50 to PCS100, whereas ABTS activity showed no significant difference between the two formulations. This difference may reflect the distinct radical systems and assay conditions used in the two methods. Accordingly, the results are interpreted as assay-dependent responses; the values from the two assays should not be treated as directly interchangeable.

Although some antioxidant values for the PCS and enriched Turkish delight appear numerically similar, the results are expressed on different bases (per 100 mL and per 100 g) and were obtained from different matrices. They should therefore not be interpreted as directly equivalent concentrations or as proof of complete retention. The assays indicate that antioxidant activity was detectable in the enriched products under the conditions used.

PCS addition enhanced the total phenolic content and antioxidant activity, confirming its potential to improve the health-promoting functional properties of Turkish delight. The markedly higher total phenolic content and antioxidant activity observed in both PCS-containing formulations demonstrate the effective incorporation of PCS-derived antioxidant constituents into the Turkish delight matrix.

#### 3.2.4. Texture Analysis

Among the seven TPA parameters evaluated in texture analysis, only hardness showed a statistically significant difference across formulations (Table 6).

The control group exhibited significantly greater hardness (11.47 ± 1.59 N) than the 100% PCS group (8.27 ± 0.25 N), while the 50% PCS group (9.26 ± 0.52 N) was intermediate and not significantly different from either formulation (Table 6). The progressive softening of the gel matrix with increasing PCS concentration resulted in a less rigid gel network. A similar softening effect has been reported in other herbal extract-enriched confectionery products [7]. Additionally, the lower pH of the PCS-enriched formulations may partially hydrolyze starch chains during the high-temperature cooking process, further contributing to the observed reduction in hardness. Previous studies have also demonstrated that the textural properties of Turkish delight are influenced by formulation, particularly by the type and concentration of incorporated ingredients [3,18].

Adhesiveness, springiness, cohesiveness, gumminess, chewiness, and resilience values were not significantly affected by PCS incorporation (*p* > 0.05 for all). The near-identical springiness values across all groups confirm that the characteristic elastic recovery—which is a defining quality attribute of Turkish delight—was maintained regardless of PCS level. Similarly, the comparable cohesiveness values across groups indicate that the internal structural integrity of the gel was not disrupted by PCS incorporation. Overall, the TPA results indicate that PCS incorporation into the Turkish delight formulation induced a moderate and technologically acceptable softening of the samples without compromising the characteristic chewy, elastic texture that defines quality Turkish delight.

#### 3.2.5. HMF Analysis

HMF content increased significantly with increasing PCS concentration (Table 7). HMF values ranged from 13.13 ± 0.68 mg/kg in the control to 15.38 ± 1.26 mg/kg in the 50% PCS and 17.57 ± 0.31 mg/kg in the 100% PCS formulation.

The increase in HMF content with PCS incorporation may reflect both the introduction of HMF already present in the PCS ingredient (9.82 ± 1.51 mg/kg) and its formation during cooking. The lower pH of the enriched formulations may also have contributed to HMF formation; however, the relative contributions of these factors were not determined. A thesis study reported HMF concentrations ranging from 4.54 to 1101.58 mg/kg in traditionally produced pinecone molasses samples analyzed by HPLC [28]. Although the values measured in the present study were toward the lower end of that range, the molasses samples had substantially higher soluble solids contents (64.63–78.68 °Brix), limiting direct comparison. This broad variability nevertheless emphasizes the importance of controlling formulation and processing conditions in pinecone-derived products. Such comparisons provide context for the measured HMF levels but do not establish the safety of the Turkish delight formulations.

#### 3.2.6. FT-IR Analysis

**Figure 4:**
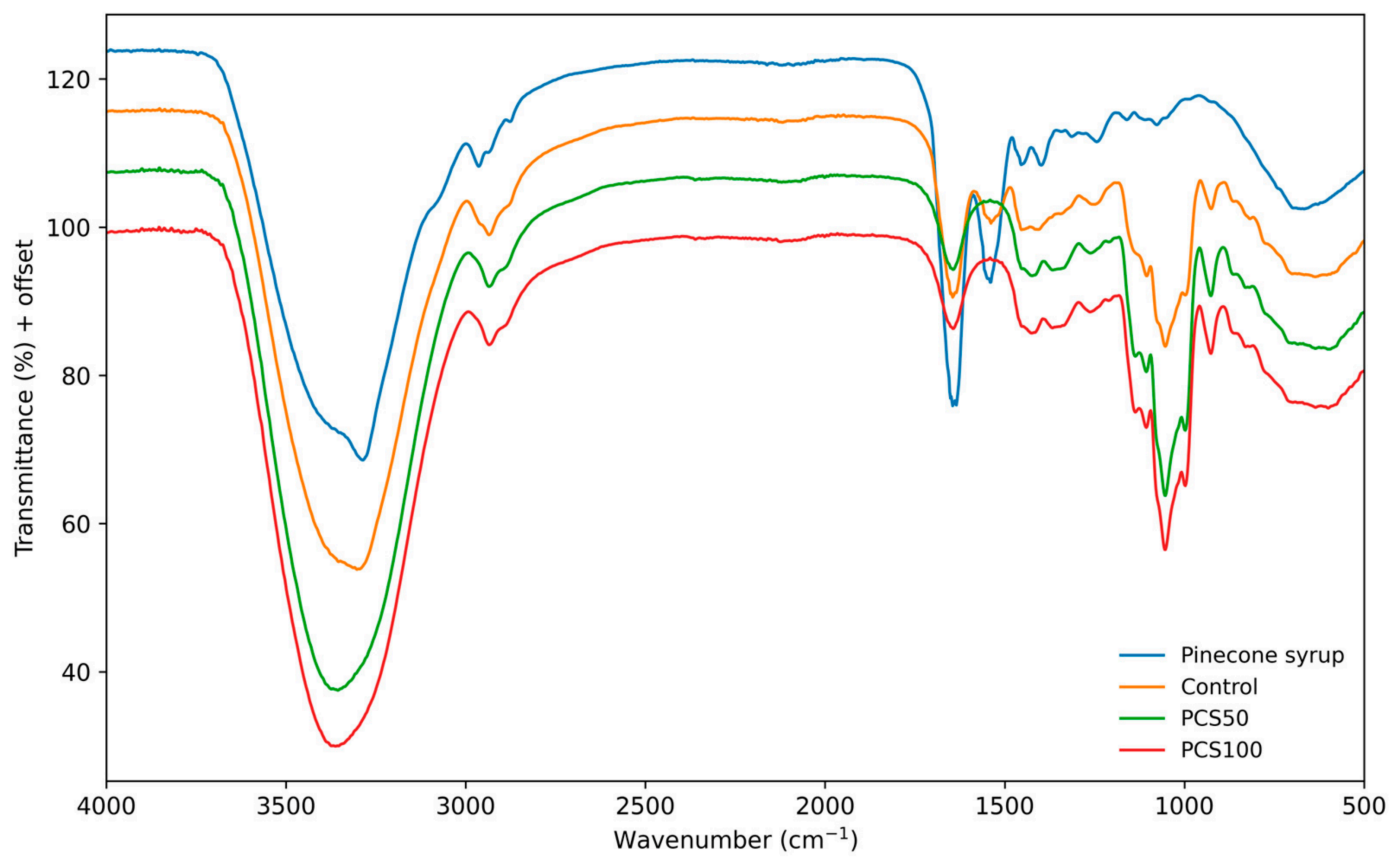
FT-IR spectra of pinecone syrup (PCS) and Turkish delight formulations.

FT-IR analysis was conducted to investigate the structural changes induced by PCS incorporation into the Turkish delight matrix and to examine potential interactions between PCS-derived phenolic compounds and the starch-sugar gel network. Representative absorbance spectra were recorded across the mid-infrared range (400–4000 cm^−1^) but the spectra are presented as transmittance (%) with vertical offsets for clarity (Figure 4).

The FT-IR spectra of pinecone syrup (PCS) and Turkish delight formulations showed generally similar profiles, although differences in band intensity and position were observed following PCS incorporation (Figure 4). A broad absorption band in the 3200–3500 cm^−1^ region was evident in all samples and can primarily be attributed to O–H stretching vibrations of water and hydroxyl-rich carbohydrate components. Changes in this region in PCS50 and PCS100 compared with the control may indicate alterations in the hydrogen-bonding environment of the starch–sugar–water matrix.

A pronounced band was also observed around 1600–1650 cm^−1^, particularly in PCS, which may be associated with H–O–H bending and overlapping vibrations of plant-derived constituents. In addition, given the high total phenolic content of PCS, C=C stretching vibrations of aromatic rings associated with phenolic compounds may also contribute to absorptions in the 1500–1600 cm^−1^ region. Such aromatic C=C ring stretching modes have been reported in the infrared spectra of phenolic-rich plant extracts and pine-derived products [19], and their potential contribution to the spectral profile of PCS and the enriched Turkish delight formulations should be considered when interpreting bands in this region. The most noticeable differences among the Turkish delight samples occurred in the 900–1200 cm^−1^ fingerprint region, where bands associated with C–O and C–O–C stretching of carbohydrates were prominent. In particular, the spectral response around 1050 cm^−1^ was more pronounced in PCS50 and PCS100 than in the control, suggesting changes in the molecular environment of carbohydrate-related functional groups following PCS incorporation.

Related spectral features have been reported for other cone-derived preparations. Çetinkaya et al. [19] observed O–H stretching bands at approximately 3309.7 and 3271.4 cm^−1^ and water-related bending bands at approximately 1638 and 1642.9 cm^−1^ in fir cone syrup and molasses, respectively. They also reported prominent carbohydrate-associated bands in the 1200–950 cm^−1^ region of the sugar-containing molasses. These observations provide a comparative basis for the hydroxyl- and carbohydrate-associated regions identified in the present study. However, differences in botanical source, sugar addition, and processing conditions limit direct comparison, and similarities in these regions do not independently demonstrate specific interactions between PCS phenolics and starch.

Overall, PCS50 and PCS100 formulations exhibited similar spectral profiles, and PCS incorporation mainly altered the intensity and position of existing bands rather than generating new characteristic bands. These findings suggest that PCS was incorporated into the Turkish delight matrix primarily through intermolecular interactions, including hydrogen bonding, without substantial alteration of the main functional-group profile.

The similar FT-IR profiles do not necessarily indicate an absence of phenolic constituents. The starch- and sugar-rich matrix contributes prominent carbohydrate-related bands that may overlap with signals from minor plant-derived components. Although total phenolic content increased after ingredient incorporation, FT-IR analysis alone cannot identify individual phenolics or establish specific aromatic structures in the final product. The observed spectral changes are therefore interpreted cautiously.

#### 3.2.7. Sensory Analysis

**Figure 5:**
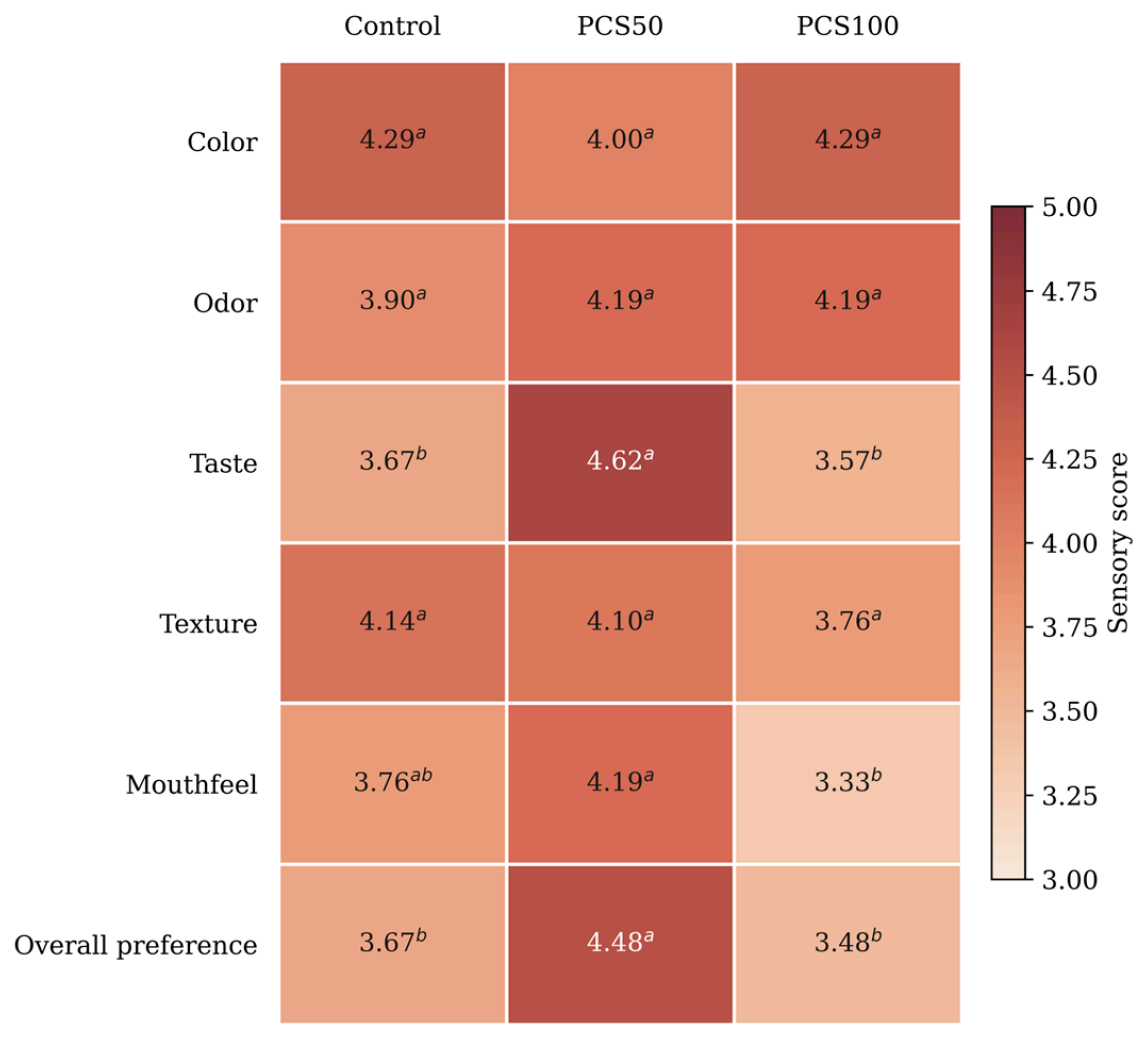
Heatmap visualization of sensory scores of Turkish delight formulations. Different superscript letters within each sensory attribute indicate significant differences among formulations (Tukey HSD, *p* < 0.05).

The sensory profiles of the Turkish delight formulations evaluated across six key attributes (color, odor, taste, texture, mouthfeel, overall preference) are presented in the heatmap (Figure 5). Figure 5 shows that the incorporation of PCS affected the sensory characteristics of Turkish delight samples differently depending on the level of substitution. The figure demonstrates that 50% PCS generally maintained or improved the sensory profile compared with the control sample, particularly for taste, odor, mouthfeel, and overall preference. In contrast, increasing the PCS level to 100% did not provide a further sensory advantage and resulted in lower scores for several attributes.

Statistical analysis revealed no significant differences among the samples regarding color, odor and texture (Figure 5). The color scores remained consistently high across all batches (4.00–4.29), suggesting that the incorporation of PCS did not adversely mask or degrade visual appeal. However, although it was not significant, 50% PCS-added samples had a slightly lower score.

Similarly, the comparable odor scores (3.90–4.19) suggest that the resinous aroma associated with PCS did not adversely affect odor acceptability at either incorporation level. In contrast, taste was strongly influenced by the PCS level (*p* < 0.001). 50% PCS received the highest taste score (4.62), significantly exceeding both the control (3.67) and 100% PCS (3.57), whereas the latter two samples did not differ significantly from each other. This finding suggests that moderate incorporation of PCS provided a desirable flavor contribution to Turkish delight, whereas complete replacement did not produce the same sensory feel. A similar pattern was observed for mouthfeel. PCS50 exhibited the highest mean score (4.19), while PCS100 had the lowest score (3.33); these two formulations differed significantly (*p* < 0.05), whereas the control (3.76) occupied an intermediate position and did not differ significantly from either group.

Texture scores showed that structural elasticity and mechanical integrity of the matrix remained well preserved upon pinecone syrup addition, aligning with instrumental texture profile observations (Table 6). The decrease in hardness values in texture analysis with increasing PCS incorporation was also reflected in the sensory evaluation, where texture scores showed a similar decreasing trend, particularly in PCS100. However, the differences in sensory texture scores were not statistically significant (*p* > 0.05). These findings showed that structural elasticity and mechanical integrity of the matrix remained within acceptable limits upon pinecone syrup addition.

Ideally, plain Turkish delight should have a characteristic Turkish delight flavor without any added odor. However, in types of Turkish delight that contain flavorings, it is necessary to have both the taste of Turkish delight and the intended flavor and aroma. Flavors such as a burnt smell or a raw starch taste are undesirable attributes in terms of quality [29]. In the present study, this balance appeared to be achieved more successfully with moderate PCS incorporation. 50% PCS-incorporated formulation received the highest taste (4.62) and overall preference (4.48) scores, suggesting that the characteristic contribution of PCS was well accepted at this level. However, increasing the replacement level to 100% did not provide the same sensory benefit, indicating that a higher PCS concentration may have resulted in a less favorable balance between the characteristic Turkish delight taste and the flavor imparted by PCS. A similar distinction between antioxidant enrichment and sensory preference was reported by Batu and Arslan [31] for Turkish delight containing black grape and sour cherry syrups. Although antioxidant activity was highest at 7.5% incorporation, samples containing 2.5% received the highest scores for appearance, color, and aroma. Similarly, PCS50 was preferred over PCS100 in the present study, indicating that increasing the incorporation level does not necessarily improve sensory acceptance.

## 4. Conclusions

This study demonstrated that sugar-free pinecone syrup (PCS) can be successfully incorporated into Turkish delight formulation at substitution levels of 50% and 100%, with the potential of producing a functional confectionery product with substantially enhanced bioactive properties. Both PCS-enriched formulations exhibited substantially higher total phenolic content and in vitro antioxidant activity than plain Turkish delight, demonstrating the potential of PCS to enhance these properties in the final product. HMF content increased with PCS incorporation; however, the increase was moderate under the applied processing conditions. Increasing PCS concentration also resulted in pronounced color changes and modifications in texture, while FT-IR analysis indicated PCS–starch intermolecular interactions without major changes in the characteristic functional groups of the confectionery matrix. Color scores in sensory evaluation remained favorable across all formulations. Hardness decreased moderately with PCS incorporation, while springiness, cohesiveness, and other textural parameters were preserved, maintaining the characteristic elastic texture of the Turkish delights. Sensory evaluation revealed that the effect of PCS was not proportional to its concentration: PCS50 received significantly higher taste and overall preference scores than both the control and PCS100, while also showing favorable mouthfeel. Overall, the findings demonstrate that partial replacement of water with PCS, particularly at the 50% level, in the Turkish delight formulation provides a favorable balance between enhanced antioxidant properties and sensory acceptability. Thus, PCS represents a promising natural ingredient for the development of value-added Turkish delight with improved functional properties.

It should be noted that pinecone syrup lacks a standardized production protocol, and preparation methods vary considerably. The syrup is traditionally obtained by boiling fresh or dried pinecones with water and sugar, but the species of pine used, the cone-to-water-to-sugar ratio, and boiling duration differ widely in practice, and these variables are known to significantly influence the phytochemical profile of the final syrup, including its total phenolic content, flavonoid composition, volatile terpene fraction, pH, and color characteristics. Consequently, the physicochemical and functional properties of the PCS used in the present study reflect the specific preparation conditions applied and may not be directly comparable to syrups and/or extracts produced under different conditions. This inherent variability represents both a challenge and an opportunity for future research: standardization of pinecone syrup production—with respect to pine species, processing parameters, and target bioactive concentrations—would be a necessary prerequisite for any commercial application of PCS-enriched confectionery products, and would allow for more reproducible and comparable findings across studies. Also, future studies should characterize the mineral composition of PCS and PCS-enriched Turkish delight, including potentially toxic elements, to assess their nutritional contribution and support a more comprehensive safety evaluation.

## Figures and Tables

**Table 1:** Turkish delight formulations.

Turkish Delight Type *	Water (mL)	Pinecone Syrup (mL)	Sugar (g)	Starch (g)	Citric Acid (g)
Control	1000	-	1000	145	1.5
PCS50	500	500	1000	145	1.5
PCS100	-	1000	1000	145	1.5

* Control: plain Turkish delight; PCS50: Turkish delight containing 50% pinecone syrup; PCS100: Turkish delight containing 100% pinecone syrup.

**Table 2:** Pinecone syrup analysis results.

Parameter	Results
pH	3.76 ± 0.01
Moisture (%)	97.98 ± 0.19
°Brix	2.73 ± 0.06
Water activity (a_w_)	0.960 ± 0.002
TPC (mg GAE/100 mL)	237.72 ± 15.95
DPPH (μmol TE/100 mL)	434.78 ± 0.48
ABTS (μmol TE/100 mL)	157.98 ± 0.19
HMF (mg/kg)	9.82 ± 1.51
Color parameters	
L*	40.90 ± 0.21
a*	7.48 ± 0.08
b*	11.58 ± 0.20
C*	13.79 ± 0.21
h°	57.14 ± 0.20

Values are expressed as mean ± SD. TPC: total phenolic content; GAE: gallic acid equivalents; TE: Trolox equivalents.

**Table 3:** Physicochemical analysis results of Turkish delight samples.

Parameter	Control	PCS50	PCS100
pH	4.24 ± 0.13 ^a^	3.89 ± 0.16 ^b^	3.67 ± 0.02 ^b^
Moisture (%)	15.00 ± 0.11 ^a^	14.60 ± 0.86 ^a^	14.37 ± 0.67 ^a^
Water activity (a_w_)	0.757 ± 0.002 ^a^	0.745 ± 0.030 ^a^	0.679 ± 0.049 ^a^
Ash (%)	0.042 ± 0.010 ^b^	0.049 ± 0.019 ^ab^	0.078 ± 0.002 ^a^
Total sugar (%)	60.86 ± 0.77 ^a^	58.17 ± 2.37 ^a^	61.67 ± 1.69 ^a^

Values are expressed as mean ± SD. Values in the same row not sharing a common superscript letter differ significantly (Tukey HSD, *p* < 0.05).

**Table 4:** Color analysis results of Turkish delight samples.

Parameter	Control	PCS50	PCS100
L*	63.90 ± 3.31 ^a^	50.51 ± 2.21 ^b^	37.35 ± 0.45 ^c^
a*	−1.78 ± 0.18 ^b^	12.93 ± 0.31 ^a^	13.97 ± 1.04 ^a^
b*	12.79 ± 0.90 ^b^	24.33 ± 1.76 ^a^	10.44 ± 1.16 ^b^
C*	12.92 ± 0.88 ^b^	27.56 ± 1.42 ^a^	17.44 ± 1.53 ^b^
h° (°)	97.95 ± 1.14 ^a^	61.94 ± 2.23 ^b^	36.71 ± 1.04 ^c^
ΔE*	-	23.11 ± 0.72 ^b^	30.99 ± 0.74 ^a^

Values are expressed as mean ± SD. ΔE*: total color difference calculated relative to the control sample. Different superscript letters within the same row denote significant differences among Turkish delight formulations (Tukey HSD, *p* < 0.05).

**Table 5:** Total phenolic content and antioxidant activity results of Turkish delight samples.

Parameter	Control	PCS50	PCS100
TPC (mg GAE/100 g)	7.3 ± 1.1 ^c^	130.1 ± 9.0 ^b^	208.6 ± 5.2 ^a^
DPPH (μmol TE/100 g)	5.1 ± 1.0 ^c^	417.6 ± 9.8 ^b^	435.6 ± 0.9 ^a^
ABTS (μmol TE/100 g)	11.3 ± 7.2 ^b^	158.5 ± 1.1 ^a^	157.7 ± 1.0 ^a^

Values are expressed as mean ± SD. Different superscript letters within the same row denote significant differences among Turkish delight formulations (Tukey HSD, *p* < 0.05).

**Table 6:** TPA results of Turkish delight samples.

Parameter	Control	PCS50	PCS100
Hardness (N)	11.47 ± 1.59 ^a^	9.26 ± 0.52 ^ab^	8.27 ± 0.25 ^b^
Adhesiveness (N·s)	−7.86 ± 4.69 ^a^	−10.34 ± 0.83 ^a^	−10.87 ± 3.05 ^a^
Springiness	0.837 ± 0.062 ^a^	0.834 ± 0.016 ^a^	0.841 ± 0.028 ^a^
Cohesiveness	0.473 ± 0.083 ^a^	0.478 ± 0.056 ^a^	0.494 ± 0.030 ^a^
Gumminess (N)	5.51 ± 1.74 ^a^	4.44 ± 0.73 ^a^	4.10 ± 0.37 ^a^
Chewiness (N)	4.68 ± 1.83 ^a^	3.70 ± 0.56 ^a^	3.44 ± 0.23 ^a^
Resilience	0.086 ± 0.006 ^a^	0.101 ± 0.003 ^a^	0.092 ± 0.018 ^a^

Values are expressed as mean ± SD. Different superscript letters within the same row indicate significant differences (Tukey HSD, *p* < 0.05).

**Table 7:** HMF results of Turkish delight samples.

Parameter	Control	PCS50	PCS100
HMF mg/kg	13.13 ± 0.68 ^a^	15.38 ± 1.26 ^b^	17.57 ± 0.31 ^c^

Values are expressed as mean ± SD. Different superscript letters within the same row indicate significant differences (Tukey HSD, *p* < 0.05).

## Data Availability

The original contributions presented in this study are included in the article. Further inquiries can be directed to the corresponding author.

## Abbreviations

The following abbreviations are used in this manuscript:

C*	Chroma
GAE	Gallic acid equivalents
h°	Hue angle
HMF	Hydroxymethylfurfural
PCS	Pinecone syrup
PCS50	Turkish delight containing 50% pinecone syrup
PCS100	Turkish delight containing 100% pinecone syrup
SD	Standard deviation
TE	Trolox equivalents
TPC	Total phenolic content
